# *In Silico* Analysis of a Novel Plasmid from the Coral Pathogen *Vibrio* *coralliilyticus* Reveals Two Potential “Ecological Islands”

**DOI:** 10.3390/microorganisms4010003

**Published:** 2016-01-04

**Authors:** Jenny Wachter, Stuart A. Hill

**Affiliations:** Department of Biological Sciences, Northern Illinois University, DeKalb, IL 60115, USA; jenmccono@gmail.com

**Keywords:** plasmid, annotation, ecological islands, pathogenicity

## Abstract

As virulence often correlates with the presence of plasmid replicons in several *Vibrio* spp., this study investigated whether non-chromosomal DNA could be found in the coral pathogen, *Vibrio coralliilyticus* BAA-450. A circular plasmid, 26,631 bp in size, was identified. DNA sequence analysis indicated that the plasmid contained 30 open reading frames, six tRNA genes, 12 inverted repeats, three direct repeats and presented no continuous sequence identity to other replicons within the database. Consequently, these findings indicate that this is a novel, previously unidentified, plasmid. Two putative “ecological islands” were also identified and are predicted to encode for various factors that would facilitate growth and survival under different ecological conditions. In addition, two open reading frames may encode proteins that contribute to the pathogenicity of the organism. Functional cooperativity is also indicated between several plasmid- and chromosomally-encoded proteins, which, in a single instance, would allow a fully functioning nutrient uptake system to be established.

## 1. Introduction

*Vibrio coralliilyticus* has been identified as a major infectious agent causing tissue damage in scleratinian corals located in the Indian Ocean, the Red Sea and throughout the Indo-Pacific; in soft corals from the Mediterranean Sea; in fish and oyster larvae along the British coastline; and, in bivalve larvae from Brazil. Consequently, it is regarded as a global marine pathogen [1,2]. *V. coralliilyticus* initiates a coral infection by chemotaxically moving towards the mucus secreted by healthy *P. damicornis* corals and adhering to the coral tissue through the expression of β-galactosidase-containing adhesins [3]. The secretion of two enzymes, superoxide dismutase and catalase, detoxifies the oxygen radicals produced during zooxanthellae photosynthesis and potentiates the survival of the organism [4]. Infection not only causes coral bleaching but can also damage the coral tissue itself causing the condition known as white syndrome pathology [5,6]. White syndrome occurs in various locations within the Indo-Pacific region and only affects scleratinian corals, with advanced tissue necrosis ultimately leading to coral death [6].

Plasmids, as well as filamentous bacteriophage, are common among *Vibrio*s and often provide an organism with virulence or growth factors [7,8,9]. The shrimp pathogen *V. nigripulchritudo* contains two plasmids which contribute to virulence, with the smaller being almost identical to the *V. shilonii* plasmid, pAK-1 [8]. *Vibrio* plasmids also contain genes that aid in the survival of the cell. For example, pJM1 of *V. angruillarum* encodes an iron sequestering system [7], whereas other plasmids may encode proteins that facilitate organic nutrient degradation, nutrient acquisition, as well as confer heavy metal resistance [10].

As plasmids are known to be instrumental in the emergence of certain pathogenic *Vibrio* strains, this study focused on identifying and annotating a novel plasmid in the global marine pathogen *V. coralliilyticus* BAA-450. Currently, five *V. coralliilyticus* genome sequences are in the NCBI data base with three mega-plasmids (approximately 300 kb or larger) having been identified [11,12,13,14,15]. The plasmid identified in this study is much smaller and would appear to be unique to the *V. coralliilyticus* BAA-450 strain as BLAST analysis against these five genome sequences did not reveal any matches. Annotation and *in silico* analysis has revealed several potential proteins predicted to be encoded from the plasmid that might confer properties on the organism that may facilitate survival within unique environments.

## 2. Experimental Studies

### 2.1. Bacterial Strains and Growth Conditions

*Vibrio coralliilyticus* BAA-450 was obtained from ATCC (Manassas, VA, USA). *Eschericia coli* DH5α (Gibco BRL: Gaithersburg, MD, USA) was used for all cloning experiments. *V. coralliilyticus* BAA-450 was propagated in Heart Infusion broth (HI) (BD Medical, Downer’s Grove, IL, USA) that included 2% (*w*/*v*) NaCl. Stock cultures of *V. coralliilyticus* BAA-450 were maintained every 2–3 days on HI plates following overnight incubation at 30° C. All liquid growth experiments were performed using 100 mL cultures at 30 °C with shaking at 150 rpm. *E. coli* was grown using Luria-Bertani (LB) medium at 37 °C. Where appropriate, the LB medium was supplemented with 100 µg/mL carbenicillin/ampicillin (Sigma: St. Louis, MO, USA).

### 2.2. Vibrio Coralliilyticus Plasmid Isolation

For large-scale plasmid isolation from *V. coralliilyticus* BAA-450, a 100 mL culture was incubated for 12 h, with plasmid DNA being released by alkaline lysis. The aqueous fraction was further extracted with phenol/chloroform (1:1) prior to plasmid precipitation with 100% ethanol [16]. The resulting plasmid pellets were resuspended in 50 µL of TEN buffer (10 mM Tris-Hcl, 10 mM EDTA, 150 mM NaCl).

### 2.3. Molecular Biology Protocols

Standard molecular biological protocols were used during the study. Polymerase chain reaction (PCR) experiments were performed using an Applied Biosystems 2720 Thermal Cycler (Singapore). For 1–4.5 kb amplicon fragments, GoTaq^®^ Flexi DNA Polymerase was used (Promega: Madison, WI, USA) according to the manufacturer’s directions; for 4–10 kb fragments, PrimeSTAR HS DNA Polymerase (TaKaRa: Otsu, Japan) was used as per the manufacturer’s directions. Usually, GoTaq^®^ PCR reactions ran for 30 cycles with annealing temperatures set in accordance with the predicted melting temperature of the oligonucleotides; PrimeSTAR PCR reactions typically involved 35 cycles that consisted of a 10 s. denaturing step at 98 °C, an annealing temperature of 63 °C for 5 s, and an elongation time of 1 min per kilobase at 72 °C.

### 2.4. Vibrio coralliilyticus BAA-450 Plasmid Sequencing

Purified *V. coralliilyticus* BAA-450 plasmid was digested with various restriction enzymes with the DNA fragments being cloned into pBluescript allowing PCR amplicons to be generated using the M13 universal primers; the amplicons then underwent dideoxy sequencing (Macrogen: Seoul, Korea). DNA sequence was analyzed using Geneious software [17]. Based on the sequence data from 38 individual fragments, custom primers were then designed to amplify novel *V. coralliilyticus* BAA-450 plasmid sequences (Table S1). A total of 115 DNA fragments were sequenced which provided coverage of the entire plasmid. All DNA sequences were initially assessed at the nucleotide level by BLAST analysis against the *V. shilonii* pAK-1 plasmid in the NCBI database [18]. Following the alignment of the unique *V. coralliilyticus* plasmid DNA sequence using the Geneious software, open reading frames (*orfs*) were identified using the NCBI ORF finder [19]. The predicted *orfs* were then assessed by BLAST analysis against the NCBI database using the blastp program [18]. Alignments were translated using the Show Translation program; the percentage of adenine and thymine bases (AT) and codon usage patterns were calculated using DNA STATS and Codon Usage available on The Sequence Manipulation Suite [20]. Enzyme restriction sites were identified with the NEBcutter V2.0 tool made available through New England Biolabs [21]. Putative promoters for each *orf* were identified using BPROM available on Softberry [22]. tRNAs were identified using the tRNAscan-SE program [23]. Inverted repeats and tandem repeats were identified using EMBOSS explorers’ inverted program [24]. Insertion sequence elements were identified using the IS Finder database search tool [25]. Protein alignments were constructed using Clustal Omega available through the European Bioinformatics Institute [26]. The RefSeq ID of the *Vibrio coralliilyticus* plasmid is NC_020451.1

## 3. Results and Discussion

### 3.1. Vibrio Coralliilyticus BAA-450 Plasmid

**Figure 1:**
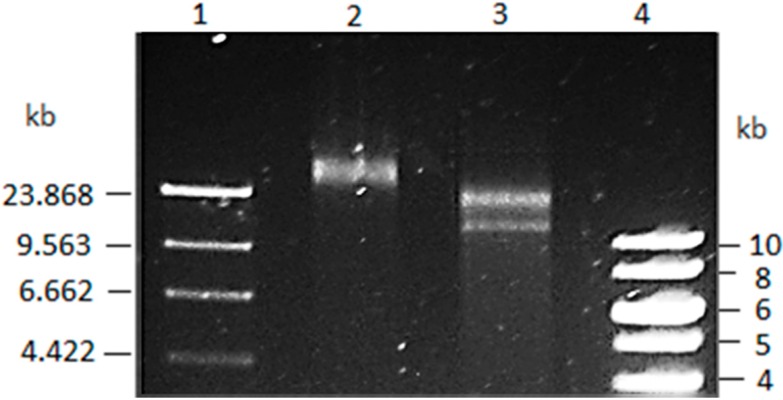
Isolation of the BAA-450 plasmid Lane 1: λ DNA digested with *Hin*dIII; Lane 2: *V. coralliilyticus* Plasmid digested with *Bgl*I; Lane 3: *V. coralliilyticus* Plasmid *Sac*II; Lane 4: Thermo Scientific™ GeneRuler 1 kb Ladder.

The purified *V. coralliilyticus* BAA-450 plasmid was initially assessed by restriction analysis where a linearized molecule in excess of 23 kb was identified (Figure 1). Following DNA sequencing of 115 useable fragments, a circular plasmid, 26,631 bp in length, was assembled. The initial BLAST analysis against *V. shilonii* pAK-1 plasmid revealed no sequence similarity, with subsequent BLAST analyses being unable to uncover any significant continuous sequence similarity with any DNA sequence within the NCBI database. Consequently, the BAA-450 plasmid appeared to be unique to *V. coralliilyticus*. The circularity of the plasmid was determined by restriction analysis using the derived DNA sequence as template; *Bgl*I digestion (a predicted single cutter) yielded a single band of appropriate size (26,631 bp), whereas *Sac*II (a predicted double cutter) yielded two bands (14,316 and 12,315 bp).

### 3.2. Annotation and Identification of Plasmid-Encoded Orfs

**Figure 2:**
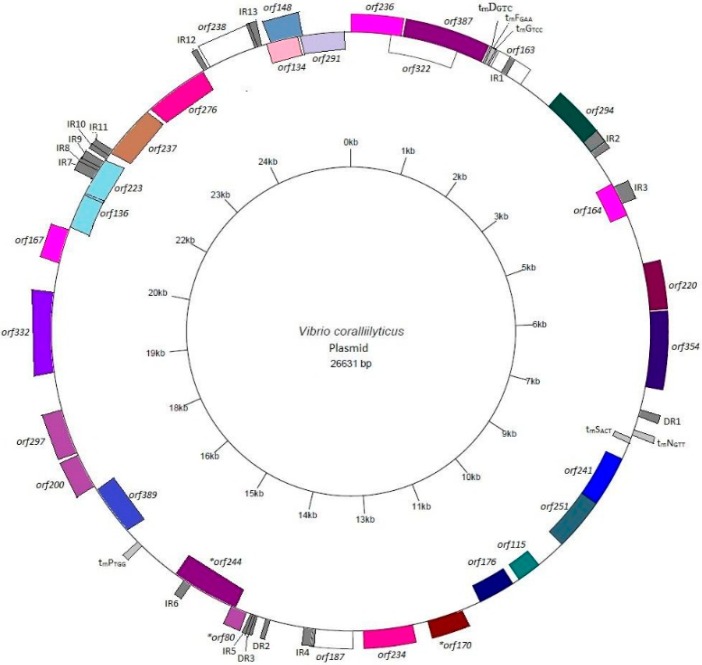
Linkage map of the BAA-450 plasmid. Following DNA sequencing, the identified *orfs* were assembled and their locations on the plasmid are displayed using [27]. The shading of the *orfs* corresponds to the putative proteins they encode and are as follows: transporter/permease proteins are shaded pink; transposases are shaded purple; ligase is shaded green; synthase is shaded blue; phosphorylase is shaded a dull green; decarboxylase is shaded a lime green; carboxykinase is shaded a dark blue; mobilization, relaxase and replication *orfs* are shaded plum purple; reductase is shaded a dusty blue; glycosyltransferase is shaded gold; and the *orf* encoding a hypothetical protein is white. tRNAs, indirect repeats (IR), and direct repeats (DR) are shown in gray.

The derived DNA sequence was annotated and the complete linkage map is presented in Figure 2. Most of the putative *orfs* that were identified began with the ATG codon. However, one putative *orf* (*orf80*) was identified that began with a TTG initiation codon. Additionally, as some of the predicted *orfs* overlapped one another, expression of transcripts arising from these *orfs* was analyzed with quantitative real time PCR (qRT-PCR) using primers that were outside of the overlapped regions. Overlapping *orfs* whose transcripts were not detected through qRT-PCR were not included in further analysis (data not shown). However, several overlapping *orfs* (*orf80, orf134,* and *orf236*) could not be analyzed through qRT-PCR due to an erroneously high background. Nonetheless, due to computational probability, these overlapping *orfs* were included in subsequent analysis although their validity has not been confirmed through experimental data. Transcripts were detected through qRT-PCR analysis from all other overlapping *orfs* (*orf148, orf291, orf287,* and *orf244*) (data not shown)*.* A complete listing of the putative *orf*s identified by BLASTP analysis, their location on the plasmid and their hypothetical protein functions can be found in Table 1. Predicted promoter elements, as well as the identification of potential operons, are presented in Tables S2 and S3, respectively.

### 3.3. Identification of Potential “Ecological” Islands within the Plasmid Replicon

**Figure 3:**
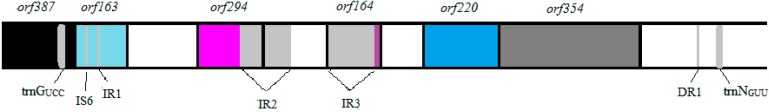
The region between *trn*G_UCC_ and *trn*N_GUU_ in the BAA-450 plasmid. This 6111 bp fragment contains five *orfs*, including *orf387* which encodes a putative transposase that may have been instrumental in the insertion of this fragment into the plasmid (not drawn to scale).

**Figure 4:**
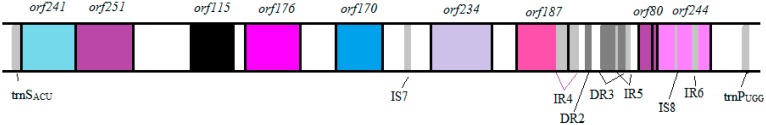
The region between *trn*P_UGG_ and *trn*S_ACU_ in the BAA-450 plasmid. This 7052 bp fragment contains eight *orfs*, including *orf244* which encodes a putative transposase enzyme that may have been involved in the incorporation of this fragment into the plasmid (not drawn to scale).

Analysis of the BAA-450 plasmid also revealed the presence of several miscellaneous features that included the presence of six tRNA genes (Table 2), eight putative insertion sequences (IS) (Table S4), 12 inverted repeats (IR) (Table S5), and three direct repeats (Table S6). In pathogenic bacteria, tRNA genes often flank pathogenicity islands, with the tRNA loci being the target sites for integration of horizontally acquired foreign DNA [8,9,28,29]. In the BAA-450 plasmid, the region of DNA between *trn*G_UCC_ and *trn*N_GUU_ contains five *orf*s, with the *trn*G_UCC_ gene being located within *orf387*. Interestingly, *orf387* encodes a putative transposase which may have been instrumental in the insertion of this 6111 bp segment of DNA into the plasmid (Figure 3). Similarly, eight *orf*s were found between *trn*P_UGG_ and *trn*S_ACU_, including *orf244,* which is again believed to encode a transposase and could explain the presence of this 7,052 bp DNA fragment (Figure 4). Assessment of the guanine and cytosine base (GC) content between the BAA-450 plasmid and the genome indicated a significant difference, which is more noticeably apparent when the DNA encoding the tRNA segments is removed from the calculation (Table 3). Furthermore, a comparison of the plasmid-located tRNA genes to those present on the *V. coralliilyticus* BAA-450 genome also shows a much lower GC composition indicating that the plasmid tRNA genes were not acquired from the chromosome. Collectively, these observations suggest that these two stretches of DNA located between flanking tRNA genes may constitute “ecological” islands that were horizontally acquired.

**Table 1:** Putative *orf*s within the *V. coralliilyticus* plasmid.

Name	Starting and Ending Base	Length (bp)	Reading Frame	Putative Protein	e-Value	% AT
*orf236*	1–711	711	+1	ABC transporter, ATP-binding protein LivF-like	3 × 10^-17^	57
*orf387*	742–1905	1164	+1	transposase, IS4	6 × 10^-123^	43
*orf163*	2058–2549	492	+3	Na^+^/H^+^ antiporter	2 × 10^-65^	59
*orf294*	3053–3937	885	+2	Biotin-protein ligase	2 × 10^-46^	59
*orf164*	4967–4473	495	−2	branched-chain amino acid ABC transporter permease protein, LivM-like	2 × 10^-24^	62
*orf220*	5694–6356	663	+3	oligoendopeptidase F	8 × 10^−80^	62
*orf354*	6380–7444	1065	+2	Trk transporter membrane-spanning protein-K^+^ transport	9 × 10^−103^	62
*orf241*	9244–8519	726	−3	methylglyoxyl synthase	9 × 10^−68^	58
*orf251*	10,005–9250	756	−1	purine nucleoside phophorylase	7 × 10^−73^	64
*orf115*	10,790–10,443	348	−2	oxaloacetate decarboxylase α-chain	3 × 10^−34^	61
*orf176*	11,426–10,896	531	−2	phosphoenolpyruvate carboxykinase	4 × 10^−44^	65
*orf142*	11,793–12,218	426	+3	maltose/maltodextrin ABC transporter, permease protein	4 × 10^−19^	57
*orf234*	12,438–13,142	705	+3	ykvW heavy metal-(Cd/Co/Hg/Pb/Zn)-translocating P-type ATPase	1 × 10^−39^	62
*orf187*	13,290–13,853	564	+3	mobilization protein	3 × 10^−18^	59
orf80 *	14,423–14,662	240	+2	RepE replication protein	2 × 10^−15^	73
*orf213*	15,225–14,587	639	−1	transposase	1 × 10^−35^	65
*orf389*	17,190–16,021	1170	−1	glutathione reductase	6 × 10^−104^	58
*orf200*	17,234–17,836	603	+2	replication protein	6 × 10^−29^	67
*orf297*	17,904–18,797	894	+3	DNA replication protein, putative	1 × 10^−36^	62
				Clp protease protein	5 × 10^−32^	
*orf332*	19,200–20,198	999	+3	maltodextrin-binding protein MdxE	3 × 10^−129^	58
*orf167*	20,696–21,199	504	+2	branched-chain amino acid ABC transporter, permease protein, LivH-like	4 × 10^−57^	56
*orf136*	21,865–21,455	411	−3	rlx domain protein	9 × 10^−56^	57
*orf223*	22,518–21,847	672	−1	mobilization protein	1 × 10^−108^	54
*orf237*	23,324–22,611	714	−2	ABC superfamily ATP binding cassette transporter, permease protein	1 × 10^−34^	61
*orf276*	24,222–23,392	831	−1	YKVW heavy metal-(Cd/Co/Hg/Pb/Zn)-translocating P-type ATPase	7 × 10^−51^	63
*orf238*	24,465–25,181	717	+3	hypothetical protein CAT7 09585	4 × 10^−91^	60
*orf148*	25,392–25,838	447	+3	PTS system, IIC component	5 × 10^−22^	61
*orf134*	25,636–25,232	405	−3	glycosyltransferase	6 × 10^−21^	62
*orf291*	26,533–25,658	876	−3	ABC exporter, membrane-spanning/permease subunit	2 × 10^−143^	62

* Begins with TTG initiation codon.

**Table 2:** Putative tRNAs Encoded in the *V. Coralliilyticus* Plasmid.

Name	Starting and Ending Base	Length	Amino Acid	Codon
*trn*D_GUC_	1807–1879	73	Aspartic acid	GAC
*trn*F_GAA_	1896–1968	73	Phenylalanine	TTC
*trn*G_UCC_	1979–2049	71	Glycine	GGA
*trn*N_GUU_	8159–8230	72	Asparagine	AAC
*trn*P_UGG_	15,608–1,5681	74	Proline	CCA
*trn*S_ACU_	8557–8484	74	Serine	AGT

**Table 3:** GC content of the *V. coralliilyticus* BAA-450 plasmid and genome.

*Vibrio coralliilyticus*	Length (bp)	% GC Content
Entire Plasmid	26,631	40
Plasmid without tRNA segments	13,472	42
Segment between *trn*G_UCC_ and *trn*N_GUU_	6111	41
Segment between *trn*P_UGG_ and *trn*S_ACU_	7052	37
Genome	5,680,628	45

Genome data for *V. coralliilyticus* BAA-450 genome was obtained from the NCBI database.

Fourteen putative proteins are found within the proposed "ecological" islands. Of these, four have predicted properties that would confer an ecological advantage. Analysis of ORF163 identified a multi-domain NhaC (sodium/hydrogen antiporter) component between amino acid residues 89–143. Significant sequence homology was shared between ORF163 and a Na^+^/H^+^ antiporter of *Carnobacterium* sp. 17-4 (e-value of 2 × 10^−65^) with the majority of the amino acids (1–144) aligning with the *Carnobacterium* protein. While the Na^+^/H^+^ antiporter of *Carnobacterium* sp. 17-4 is a more complex 567 amino acid protein, the presence of the NhaC domain within ORF163 infers a role for ORF163 in energy production/conversion utilizing a Na^+^/H^+^ antiporter.

Within ORF241 a MGS-like (methylglyoxal synthase) superfamily domain was identified that incorporates an active site, catalytic residues and the appropriate dimer interfaces within amino acids 18–129. This putative protein also contains a P-loop NTPase multi-domain with an ATP binding site between amino acids 154–220. A blastp search identified high sequence homology (e-value of 9 × 10^−68^) with the *Carnobacterium* sp. 17-4 MGS with the majority (amino acids 7–139) aligning with ORF241, inferring a methylglyoxal synthase role for *orf241.* A methylglyoxal synthase (151 amino acids) is also encoded within the *V. coralliilyticus* genome and shares sequence homology (e-value: 8 × 10^−35^) to ORF241 as well as containing a similar conserved domain. However, no nucleotide homology is shared between the two genes indicating that *orf241* did not originate from the genome. Consequently, ORF241 may provide the bacterium with an alternative to triosephosphate isomerase for metabolizing dihydroxyacetone phosphate that is produced during glycolysis.

ORF251 contains a DUF1250 superfamily (unknown function) domain (amino acids 1–69), a PNP UDP 1 phosphorylase domain, and a deoD purine nucleoside phosphorylase domain within amino acids 82–215. Blastp analysis identified a *Carnobacterium* sp. 17-4 purine nucleoside phosphorylase of similar length (236 amino acids) with significant sequence homology (e-value of 7 × 10^−73^) to ORF251. Consequently, *orf251* may encode a putative purine nucleoside phosphorylase *deoD* which could provide flexibility with nucleic acid metabolism.

Although the open reading frames are located 10,250 nucleotides apart, two putative proteins, ORF234 (a proposed “ecological” island protein) and ORF276, were found to contain the same conserved domains and highly similar amino acid sequences (amino acids 166–276 in ORF276 and amino acids 1–110 in ORF234). ORF276 contains a HAD-like (haloacid dehalogenase) superfamily from amino acids 156-246 plus a ZntA cation transport ATPase multi-domain encompassing aa 46–271. The HAD-like superfamily of ORF234 is located between amino acids 20–88 with the ZntA multi-domain from amino acids 2–173. Both ORF276 and ORF234 also share significant sequence similarities (e-values: 1 × 10^−149^ ORF276; 2 × 10^−88^, ORF234) with a 641 amino acids P-type ATPase cadmium transporter of *Enterococcus italicus* DSM 15952. A refined blastp search further revealed that ORF276 shared sequence homology (e-value of 7 × 10^−51^) to a YKVW 325 amino acid heavy metal-(Cd/Co/Hg/Pb/Zn)-translocating P-type ATPase from *Bacillus halodurans*, and that ORF234 shared sequence homology (e-value 1 × 10^−39^) with a 225 amino acid ykvW protein of *Bacillus cereus* ATCC 10987. Based on the presence of conserved domains and sequence homologies, it is hypothesized that both ORF234 and ORF276 function as part of a heavy metal-(Cd/Co/Hg/Pz/Zn)-translocating P-type ATPase complex.

### 3.4. Identification of Putative Pathogenicity Proteins on the BAA-450 Plasmid

ORF389 contains a PRK06116 glutathione reductase domain within amino acids 10–264 with high sequence homology (e-value of 6 × 10^−104^) to a glutathione reductase from *Salmonella enterica* subsp. *enterica serovar Typhi* str. E98-0664. However, both ORF389 and the glutathione reductase of *Salmonella* appear to lack a dimerization domain at the carboxy terminus, which could affect the functioning of these proteins as dimerization is essential for catalytic activity. Despite the truncated carboxy-terminal end, the conserved domains, and the extensive protein alignment, supports a role for ORF389 as a glutathione reductase [30]. The chromosomally encoded glutathione reductase (451 amino acids) shares sequence homology (e-value of 5 × 10^−90^) with ORF389. The chromosomal protein contains a NADB Rossmann superfamily, a PRK06116 multi-domain, plus a Pyr redox dim (Pyridine nucleotide-disulphide oxidoreductase dimerization domain) superfamily, the last domain being absent in ORF389. Partial homology (e-value of 4 × 10^−35^) was also detected between the chromosomal and plasmid nucleotide sequences encoding these proteins suggesting that the chromosomally- and plasmid-encoded proteins may indeed interact.

While ORF134 lacked various conserved domains, sequence homology (e-value of 6 × 10^−21^) was found between ORF134 and a putative glycosyltransferase enzyme (207 amino acids) from *Melissococcus plutonius* ATCC 35311. However, while sequence homology to the putative glycosyltransferase enzyme is present, the specific function of ORF134 is still uncertain since the putative protein lacks the glycosyltransferase domain which would allow for carbohydrate binding in a sugar-nucleoside disphosphate- and manganese-dependent fashion.

### 3.5. Functional Cooperativity between Putative Plasmid- and Chromosomally-Encoded Proteins

**Figure 5:**
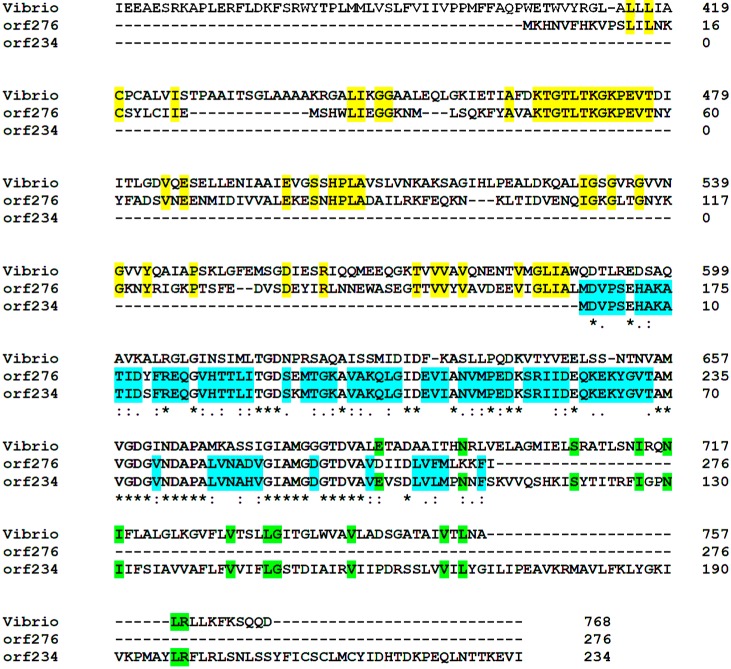
Clustal alignment of putative chromosomally-encoded and plasmid-encoded lead, cadmium, zinc and mercury transporting ATPase proteins. The alignment begins at amino acid 419 of the chromosomally-encoded protein. The gray shaded amino acids show similarity between all three proteins; the yellow shaded amino acids show similarity between ORF276 and the chromosomal protein; the green shaded amino acids show similarity between ORF234 and the chromosomal protein; and, the blue shaded amino acids show similarity between ORF276 and ORF234.

The possibility that plasmid-encoded proteins interact with chromosomally-encoded proteins was further explored following analysis of other protein homologies. The two putative plasmid-encoded heavy metal-(Cd/Co/Hg/Pb/Zn) transporter ATPase proteins (ORF276 and ORF234) were found to share functional similarity to a genomic lead, cadmium, zinc and mercury transporting ATPase protein. While the protein encoded on the chromosome is larger (759 amino acids), a portion of both plasmid putative proteins share sequence similarity to the chromosomal protein (2 × 10^−20^ for ORF234 and 2×10^−32^ for ORF276) (Figure 5). In fact, the chromosomally-encoded protein, ORF234 and ORF276 all share conserved domains that include a zinc/cadmium/mercury/lead-transporting ATPase as well as a haloacid dehalogenase superfamily. However, the DNA sequences of each of these genes share no sequence homology indicating that the plasmid genes did not originate from the chromosome. Indeed, of the 14 putative proteins located within the proposed “ecological” islands, six putative proteins (ORF294, ORF163, ORF115, ORF220, ORF241 and ORF251) shared significant homologies to chromosomally-encoded proteins suggesting functional cooperativity. However, there are some plasmid-encoded proteins which do not share sequence homology to similar chromosomally-encoded proteins. These include the putative Trk protein (ORF354), PEP carboxykinase (ORF176), glycosyltransferase (ORF134), the putative ABC exporter (ORF291), plus the putative Clp protease and replication proteins ORF80, ORF200 and ORF297).

Functional cooperativity is further indicated following analysis of the plasmid-encoded maltose/maltodextrin ABC transporter permease subunit (ORF170) and the maltodextrin-binding protein, MdxE (ORF332). In contrast, the *V. coralliilyticus* genome encodes a maltose/maltodextrin ABC transporter substrate binding protein (MalE), a maltose/maltodextrin transport ATP-binding protein (MalK), maltose/maltodextrin ABC transporter permease proteins (MalF and MalG), a maltose operon periplasmic protein (MalM), and several glycosidases. In *V. cholerae*, the homologues of these maltose/maltodextrin genes have been shown to be essential for the expression of virulence factors including cholera toxin and soluble hemaggtluninin-protease [31]. The plasmid and chromosomal maltose/maltodextrin genes may combine to form a functional maltose/maltodextrin transporter system which may play a role in the virulence of this organism.

## 4. Conclusions

The sequencing and annotation of the BAA-450 plasmid supports a potential role for the plasmid-encoded proteins in the growth and survival of *V. coralliilyticus*. Several putative *orf*s may provide factors that would protect the cell from harmful substances that may be encountered in coral ecosystems (e.g., *orf389*, which codes for a putative glutathione reductase may remove photosynthetically-derived oxygen radicals that accumulate within intracellular environments), with other proteins possibly being implicated in virulence (the putative glycosyltransferase ORF134 possibly being involved in the expression of adhesins), while others may encode various nutrient uptake systems, or, otherwise facilitate growth in high salinity environments. The DNA analysis also indicates the presence of two “ecological islands” that may have been acquired at two distinct intervals which may also facilitate growth within changing marine environments.

## Supplementary Materials

Supplementary materials can be found at www.mdpi.com/2076-2607/4/1/3/s1.
