# Supplementary material for: In Silico Analysis of a Novel Plasmid from the Coral Pathogen Vibrio coralliilyticus Reveals Two Potential “Ecological Islands”
Source: Microorganisms. 2016 Jan 4;4(1):3. doi: 10.3390/microorganisms4010003 (PMC5029508; doi:10.3390/microorganisms4010003)
Supplement: Supplementary file 1 [file microorganisms-04-00003-s001.docx]

Supplementary Materials: *In Silico* Analysis of a Novel Plasmid from the Coral Pathogen *Vibri*o *coralliilyticus* Reveals Two Potential “Ecological Islands”

Jenny Wachter and Stuart A Hill

**Table S1.** Primers utilized in this study for the sequencing of the *V. coralliilyticus* plasmid.

| **Designation** | **Nucleotide Sequence (5′–3′)** |  |
| --- | --- | --- |
| 0217vcf2 | GGA GGT TTG GAT GAG AAT TTC TAG AC |  |
| 0217vcf3 | CCC ACC CCG TCA CAA AAA G |  |
| 0217vcf4 | CAT CCT TCC TGA TCA AAT CCC TGC |  |
| 0217vcf5 | GGT TCT TAC CAC TGT ATC TTT TAC CC |  |
| 0217vcr5 | CCC AAT GAC GCG GTT CAT TTT C |  |
| 0217vcr6 | CTC AAC GGT GTA GCT CCT GG |  |
| 1clafvcf | CGC CAA CCA TTT TGC CCA AAA TG |  |
| 2clafvcf | GGT TAT GGA AAC GCA CAC GAT TTT C |  |
| 6clafvcf | CCA ACG GTG TGA ATC CCG |  |
| 11hindfvcf | CCC TCC TTT TTA GCG ACT G |  |
| 12h21pvcf | CCA GTG TGC CTA CCG GTA TC |  |
| 14pstfvcf | CCG GTT CTT CGG GAA TCG TTA AG |  |
| 16hindfvcf | GCT ACT GTT TTC GAT GCT GTT TCT AG |  |
| 2clarvcr | GAA AAT TCC GCT GGA CAA ATT TCT G |  |
| 4clafvcr | CCT TCC ATG TTG GTG TCA AAG C |  |
| 6clarvcr | CGG CTC AGG GTC GTA TGA TC |  |
| 7ecorvcr | CCA AAA AAA GAT CTC CCT CCT GC |  |
| 12h13hrvcr | CTT TTT AAA GCG TTT TTT TGC GGC |  |
| 14pstrvcr | CTG TTG GTG TGA TAC GAA TAA TGG G |  |
| 21pstrvcr | CAA TGC AGA CAA CTA CAC ACC AAG |  |
| 22xbarvcr | CGA AAG GAG ATC CAT CAC CTT G |  |
| 112h21pvcr | GCAGTCAAAACGTTGTCTATAGGC |  |
| 12vcr6 | GCTTATTCAGTGATGATTCCTGCTG |  |
| 10hindrvcr | GCTCTGTAATGATACAAAGAAGGAGC |  |
| 14pstrvcf | GCGAGTTGGAGTAGGACACAG |  |
| 10vcr6 | CGCTAGTTGCAGCACTAAAAATCC |  |
| 12vcf6 | CATAACGCTGAACGGATCAGCTG |  |
| 2916hindfvcf | GGAAACGTTCTTATCGCTTGCTC |  |
| 21pstrvcf | GGAACCAATCATTCTCTTACATCTACG |  |
| 60hindrvcf | GCT CTC TCA ACA GGT TTT CAA ATT C |  |
| 60hindrvcr | GAT TCC TTT CAT TCA TAC ACA GGA CG |  |
| 61ecorvcf | GGT GTG GTT ACC GTT TAG ACA C |  |
| 61ecofvcr | CGTGCGTAATTTGCGAATCAAACCC |  |
| 62pstrvcf | GGT AGA ACA CAA CCC TAA CGA AAT C |  |
| 62pstrvcr | CTG TTG TCC TTC CAA GTC TTC C |  |
| 65hrvcf | CCG TAA AAA TCG GTG TTA ACA ATG |  |
| 10hindrvcf | GGCTTTTGCTCACTATATGTCTCTC |  |
| 10vc2r | CGTAGAAATTCAAGTAGTGCAGCC |  |
| 37c6h12p21vcf | CAGTGAAGGACTTGTAGAAACGATG | |
| 227ecorvcr | CGGCAAAAGAGGTAAAAAGGAAGC | |

**Table S1.** *Cont.*

| 2314pstrvcf | GTGAAAGGTTCATCGTAAGCAAGAG |
| --- | --- |
| 10vcf2r | CATTTTTGCCGGATTCAAGAAATGC |
| 1716p14c2vcf | GAGCATAACACTGAAACGCTTTC |
| 4322xbafvcr | CAGACGTTAGACAAATGACGAATC |
| 54pstrvcr | CGA AGT TTT CAA AAC CGG TCC |
| 54pstrvcf | CGG ATA AAC TCT AAA TGG ATT AGC ATA AGG |
| 54pstfvcr | CAT CAC CAA ACT TGT CAT AGA GCC |
| 54pstfvcf | GGA GCA CTT TTT CTA GCA GTG TTT C |

**Table S2.** Putative σ^70^ transcriptional promoters in the *V. coralliilyticus* plasmid whose score was at or above 15 for −35 box and at or above 30 for −10 box.

| V | −35 Box | Sequence | Score | −10 Box | Sequence | Score | Promoter |
| --- | --- | --- | --- | --- | --- | --- | --- |
| *orf236* | 26,516–26,521 | TTTCCA | 42 | 26,540–26,548 | TGATCAAAT | 37 | 26555 |
| *orf387* | 643–648 | TTGAGC | 21 | 662–670 | CAGTATTTT | 42 | 673 |
| *orf163* | 1950–1955 | TCGACT | 24 | 1970–1978 | AACTAAAAT | 62 | 1985 |
| *orf294* | 3009–3017 | TTCACG | 35 | 2989–2994 | CCTTATGGT | 40 | 3024 |
| *orf241* | 9374–9369 | TTGACT ^R^ | 61 | 9350–9342 | ATCTATTTT ^R^ | 42 | 9335 |
| *orf251* | 10,111–10,106 | TTTAAT ^R^ | 36 | 10,091–10,083 | CGCTAAAAA ^R^ | 39 | 10076 |
| *orf115* | 10,872–10,867 | ATGATG ^R^ | 22 | 10,850–10,842 | CGTTATTAG ^R^ | 37 | 10835 |
| *orf234* | 12,337–12,342 | TTTCAA | 36 | 12,354–12,362 | CGTTTAAAT | 44 | 12369 |
| *orf80 ** | 14,364–14,369 | TTTACA | 47 | 14,384–14,392 | GGTTATAAT | 90 | 14399 |
| *orf244* | 15,381–15,376 | CTGCAT ^R^ | 15 | 15,363–15,355 | GCTTAAAAT ^R^ | 69 | 15348 |
| *orf389* | 17,345–17,340 | TTTCAA ^R^ | 36 | 17,327–17,319 | TTTTAGCCT ^R^ | 41 | 17311 |
| *orf332* | 19,106–19,111 | TGGAAT | 18 | 19,123–19,131 | GATCAGAAT | 33 | 19138 |
| *orf167* | 20,595–20,600 | TTAACT | 38 | 20,617–20,625 | ATATAGAAT | 53 | 20632 |
| *orf136* | 21,959–21,954 | TGGCAA ^R^ | 18 | 21,932–21,940 | TGCCAAATT ^R^ | 35 | 21925 |
| *orf237* | 23,409–23,404 | CTGAAA ^R^ | 25 | 23,388–23,380 | GGATACAAT ^R^ | 63 | 23373 |
| *orf276* | 24,279–24,274 | TAGAAA ^R^ | 23 | 24,263–24,255 | TGCAAAAAT ^R^ | 45 | 24247 |
| *orf238* | 24,321–24,326 | TTCACA | 46 | 24,340–24,348 | ACATAAAAT | 56 | 24361 |
| *orf148* | 25,226–25,231 | TTTATT | 34 | 25,246–25,254 | CAGTAAATT | 49 | 25261 |
| *orf134* | 25,692–25,687 | TTGCTG ^R^ | 47 | 25,673–25,665 | ATATATATT ^R^ | 50 | 25658 |
| *orf291* | 26,607–26,602 | TTAACA ^R^ | 43 | 26,587–26,579 | AAATATGCT ^R^ | 37 | 26571 |

* Begins with TTG initiation codon; ^R^ Reverse complement sequence.

**Table S3.** Proposed operons of the *V. coralliilyticus* plasmid.

| **Distance between *Orf*s (bp)** | ***Orf*** | **Putative Protein** |
| --- | --- | --- |
| 5 | *orf241* | methylglyoxal synthase |
|  | *orf251* | purine nucleoside phosphorylase |
| 67 | *orf200* | replication protein |
|  | *orf297* | DNA replication protein |
|  |  | clp protease protein |
| Overlap | *orf136* | rlx protein |
|  | *orf237* | mobilization protein |
| 30 | *orf236* | ABC transporter, ATP-binding protein LivF-like |
|  | *orf387* | transposase, IS4 |
| 23 | *orf220* | oligoendopeptidase F |
|  | *orf354* | Trk transporter membrane-spanning protein – K + transport |

**Table S4.** Putative IS elements located within the *V. coralliilyticus* plasmid.

| **Name** | **Starting and Ending Base** | **Length** | **IS Family** | **IS Group** | **E-Value** | **Within ORF** |
| --- | --- | --- | --- | --- | --- | --- |
| IS1 | 910–990 | 81 | IS5 | IS903 | 1×10^−16^ | ORF387 |
| IS2 | 753–872 | 120 | IS5 | IS903 | 6×10^−16^ | ORF387 |
| IS3 | 1053–1071 | 19 | IS5 | IS903 | 0.460 | ORF387 |
| IS4 | 1222–1244 | 23 | IS5 | IS903 | 0.460 | ORF387 |
| IS5 | 1357–1377 | 21 | IS5 | IS903 | 0.029 | ORF387 |
| IS6 | 2159–2181 | 23 | IS200/IS605 |  | 0.460 | ORF244 |
| IS7 | 12,328–12,351 | 24 | IS4 | IS231 | 0.120 |  |
| IS8 | 14,740–14,759 | 20 | IS1595 | IS1016 | 0.120 | ORF163 |

**Table S5.** Inverted repeats within the *V. coralliilyticus* plasmid.

| **Name** | **Starting and Ending Base** | **Length (bp)** | **Score** |
| --- | --- | --- | --- |
| IR1 | 2230–2268 | 39 | 10/10 (100%) |
| IR2 | 3781–4265 | 485 | 79/81 (97%) |
| IR3 | 4492–4907 | 416 | 125/194 (64%) |
| IR4 | 13,836–13,931 | 96 | 10/10 (100%) |
| IR5 | 14,301–14,383 | 83 | 15/15 (100%) |
| IR6 | 15,062–15,107 | 46 | 13/13 (100%) |
| IR7 | 22,085–22,123 | 39 | 12/12 (100%) |
| IR8 | 22,428–22,468 | 41 | 13/13 (100%) |
| IR9 | 22,482–22,513 | 32 | 11/11 (100%) |
| IR10 | 22,529–22,558 | 30 | 12/12 (100%) |
| IR11 | 22,578–22,618 | 41 | 12/12 (100%) |
| IR12 | 24,347–24,366 | 20 | 10/10 (100%) |
| IR13 | 25,185–25,214 | 30 | 13/13 (100%) |

Length is taken from the starting base to the ending base of the repeat; Score is taken by dividing the number of matching base pairs by the number of total bases in half of the repeat.

**Table S6.** Direct repeats found within the *V. coralliilyticus* plasmid.

| **Name** | **Starting and Ending Base** | **Length (bp)** | **Size (bp)** | **Count** | **Score** |
| --- | --- | --- | --- | --- | --- |
| DR1 | 7966–8013 | 48 | 10 | 4 | 20 (50%) |
| DR2 | 14,154–14,194 | 41 | 11 | 3 | 24 (73%) |
| DR3 | 14,260–14,362 | 103 | 22 | 4 | 51 (58%) |

Length is taken from the starting base to the ending base of the repeat; Size is the length of the repeated sequence; Count is the number of times the sequence is repeated; Score is +1 for every base match and −1 for every base mismatch.
